# Structure and Thermal Stability of ε/κ-Ga_2_O_3_ Films Deposited by Liquid-Injection MOCVD

**DOI:** 10.3390/ma16010020

**Published:** 2022-12-20

**Authors:** Edmund Dobročka, Filip Gucmann, Kristína Hušeková, Peter Nádaždy, Fedor Hrubišák, Fridrich Egyenes, Alica Rosová, Miroslav Mikolášek, Milan Ťapajna

**Affiliations:** 1 Institute of Electrical Engineering, Slovak Academy of Sciences, Dúbravská cesta 9, 841 04 Bratislava, Slovakia; 2 Faculty of Electrical Engineering and Information Technology, Institute of Electronics and Photonics, Slovak University of Technology, Ilkovičova 3, 812 19 Bratislava, Slovakia

**Keywords:** Ga_2_O_3_, liquid-injection MOCVD, thermal stability, X-ray diffraction, TEM

## Abstract

We report on crystal structure and thermal stability of epitaxial ε/κ-Ga_2_O_3_ thin films grown by liquid-injection metal–organic chemical vapor deposition (LI-MOCVD). Si-doped Ga_2_O_3_ films with a thickness of 120 nm and root mean square surface roughness of ~1 nm were grown using gallium-tetramethylheptanedionate (Ga(thd)_3_) and tetraethyl orthosilicate (TEOS) as Ga and Si precursor, respectively, on c-plane sapphire substrates at 600 °C. In particular, the possibility to discriminate between ε and κ-phase Ga_2_O_3_ using X-ray diffraction (XRD) φ-scan analysis or electron diffraction analysis using conventional TEM was investigated. It is shown that the hexagonal ε-phase can be unambiguously identified by XRD or TEM only in the case that the orthorhombic κ-phase is completely suppressed. Additionally, thermal stability of prepared ε/κ-Ga_2_O_3_ films was studied by in situ and ex situ XRD analysis and atomic force microscopy. The films were found to preserve their crystal structure at temperatures as high as 1100 °C for 5 min or annealing at 900 °C for 10 min in vacuum ambient (<1 mBar). Prolonged annealing at these temperatures led to partial transformation to β-phase Ga_2_O_3_ and possible amorphization of the films.

## 1. Introduction

Gallium oxide (Ga_2_O_3_) is an ultrawide bandgap (UWB) semiconductor material that received great research interest in the last decade due to its outstanding material properties. Its UWB (~4.5–5.3 eV) and high theoretical critical electric field (~8 MV/cm) are suitable for fabrication of high-voltage and high-power electronic devices exceeding the capabilities of current power electronic device materials (Si, GaN, and SiC) [1,2,3,4,5]. Applicability of Ga_2_O_3_ for high-power application can be well documented by Baliga figure of merit, which reaches a theoretical value of 3571 for Ga_2_O_3_, superseding its main competitors, such as GaN (667) and SiC (134) [3,4,5]. Up to now, breakdown field of 3.8 and 5.2 MV/cm was experimentally demonstrated for monoclinic (β) Ga_2_O_3_-based lateral metal oxide semiconductor field-effect transistor (MOSFET) and vertical heterostructure, respectively [6,7]. Concerning high-power switching applications, enhancement-mode β-Ga_2_O_3_ MOSFET with a power figure of merit (breakdown voltage/specific ON-state resistance) of 192.5 MW/cm^2^ was recently reported [8]. UWB of Ga_2_O_3_ makes it also very attractive for optoelectronic devices e.g., solar-blind photodetectors, or as a host material for phosphors suitable for electroluminescent displays when activated by transition metals or rare earth elements [8].

Ga_2_O_3_ crystalizes in several phases differing in bandgap and other material properties. The only thermodynamically stable phase is the monoclinic β-Ga_2_O_3_, which can be also produced as bulk crystals using melt-grown techniques [9,10]. Metastable corundum α-Ga_2_O_3_ phase offers larger bandgap, wider capabilities in forming heterostructures [11], and several µm-thick layers can be grown by a simple and scalable Mist-CVD method [12]. A metastable hexagonal structure of ε-Ga_2_O_3_ may allow for high-quality epitaxial layers grown on various hexagonal substrates, such as the often-used sapphire [13], but also GaN or SiC for enhanced heat spreading [14,15,16]. Recent studies discussed piezoelectric properties of ε-Ga_2_O_3_, which may give rise to future polarization-engineered heterostructures, similar to the case of III-N materials [17,18]. The concept of III-N and ε-Ga_2_O_3_ integration may thus offer great potential for manufacture of power transistors with lower on-state resistance. In this case, however, the thermal stability of ε-Ga_2_O_3_ during III-N barrier growth will be one of the key limiting factors and needs to be addressed.

ε-Ga_2_O_3_ belongs to the P6_3_*mc* space group in which a 4H close-packing oxygen layer sequence contains disordered Ga atoms occupying octahedra and tetrahedra sites in 2:3 stoichiometry [19,20]. However, detailed microstructural study of the films identified as ε-Ga_2_O_3_ using X-ray diffraction (XRD) analysis pointed out that the real structure of the films was composed of nanoscale domains (5–10 nm in size) with orthorhombic structure belonging to the P*na*2_1_ space group, also known as κ-phase [20]. In contrast to ε-phase, Ga atoms are ordered in the κ-phase nanodomains, occupying octahedral and tetrahedral sites where edge-sharing octahedra and the corner sharing tetrahedra form zig-zag ribbons along the [100] direction [20]. The twinned nanodomain structure results in diffraction with pseudo-hexagonal symmetry, making the discrimination between the two phases using XRD extremely challenging. This is because the probing resolution of the XRD may be lower than the ordering range of the κ-phase nanodomains, revealing the averaged, disordered structure identified as the ε-Ga_2_O_3_. As a result, standard symmetrical 2θ/ω scans are insufficient in distinguishing between ε and nanodomain κ-phase Ga_2_O_3_. On the other hand, other commonly used XRD analyses, such as φ scans, can provide more conclusive results. Yet, systematic studies on the applicability of φ scans for unambiguous discrimination between ε- and κ-phase Ga_2_O_3_ are limited. Conventional transmission electron microscopy (TEM) faces similar limitation when apertures larger than nano-sized domains for selected area electron diffraction (SAED) are used and it reaches smaller resolution than needed to distinguish lattice periodicity using phase contrast analysis. Consequently, while high-resolution TEM can be used for unambiguous identification of the κ-phase [20], conventional TEM suffers from inconclusive reciprocal lattice analysis of observed material. This is why we will refer to ε/κ-Ga_2_O_3_ rather than phase pure Ga_2_O_3_ polymorphs in the following.

There are a number of methods used for growth of ε/κ-Ga_2_O_3_, including halide vapor phase (HVPE) [15], molecular beam (MBE) [21], metal–organic vapor phase (MOVPE) epitaxy [13,21,22], pulsed laser deposition [23], and mist chemical vapor deposition (Mist-CVD) [24,25,26] on different substrates, such as sapphire, various SiC polymorphs, bulk β-Ga_2_O_3,_ and others. Recently, we demonstrated the growth of α- and β-Ga_2_O_3_ phases using liquid-injection metal–organic CVD (LI-MOCVD) on sapphire substrate [27]. In this work, LI-MOCVD was also used for growth of ε/κ-Ga_2_O_3_ on sapphire substrate.

Thermal stability represents an important concern for epitaxial films with metastable structure, as device processing typically involves high-temperature steps for e.g., Ohmic contact annealing. Thermal stability of ε/κ-Ga_2_O_3_ grown by MOCVD was examined by annealing at elevated temperatures in N_2_ and O_2_ atmospheres [14,28]. Xia et al. [14] reported ε/κ-phase stability up to 800 °C during annealing for 30 min in N_2_, while Fornari et al. [28] observed thermally stable films annealed at 700 °C for 3 h in N_2_ or O_2_ atmosphere using ex situ XRD. Detailed analysis using in situ differential scanning calorimetry revealed an onset of initial phase transformation already at 650 °C. These results demonstrate sufficiently robust thermal stability of ε/κ-Ga_2_O_3_ epitaxial films required for device processing. However, the thermal stability of ε/κ-Ga_2_O_3_ films in different conditions, such as vacuum or hydrogen, has not yet been studied.

In this work, we report on crystal structure and thermal stability of ε/κ-Ga_2_O_3_ epitaxial films grown by LI-MOCVD. In particular, we investigated the applicability of XRD or conventional TEM to discriminate between ε- and κ-phase Ga_2_O_3_ films. In addition, thermal stability of the prepared ε/κ-Ga_2_O_3_ films were examined using in situ and ex situ XRD.

## 2. Materials and Methods

Ga_2_O_3_ thin films studied here were grown by LI-MOCVD. This method represents a modification of MOCVD, where metal–organic chemicals dissolved in an appropriate solvent are injected into the vaporization part of the reactor via electromagnetic micro-valves. Thermally decomposed vapors of precursors and reactant gas are transported into the deposition part of the LI-MOCVD reactor using carrier gas, where the deposition of required material occurs on the heated substrate. The liquid-injection system offers several advantages, such as versatility of depositing materials, excellent layer thickness control via precise precursor dosing, and low vapor pressures of delivered complexes [29,30]. More details on LI-MOCVD growth of α- and β-Ga_2_O_3_ epitaxial films can be found elsewhere [27]. Si-doped Ga_2_O_3_ films with a thickness of 120 nm (measured by ellipsometry) were deposited on c-plane sapphire substrates at a deposition temperature of 600 °C. Gallium-tetramethylheptanedionate (Ga(thd)_3_) and tetraethyl orthosilicate (TEOS) as Ga and Si precursors, respectively, dissolved in toluene, and a Ar/O_2_ carrier/reactant gas flow rate of 120 and 600 sccm were used. Temperature at the vaporization part of the reactor was set to 170 °C.

The crystal structure of prepared films was studied by the XRD using the Bruker D8 DISCOVER diffractometer equipped with X-ray source with a rotating Cu anode operating at 12 kW. All measurements were performed in parallel beam geometry with a parabolic Goebel mirror in the primary beam producing the beam divergence ~0.03°. Symmetrical 2θ/ω scans were measured with the beam size of 1 × 6 mm^2^. To suppress the strong diffraction from the sapphire substrate, samples were tilted by an angle of 0.5° away from the exact diffraction position of the substrate. The azimuthal ordering of the layer structure and the orientation of the Ga_2_O_3_ lattices with respect to the sapphire substrate were analyzed by φ scans of the selected diffractions. For these measurements, the beam size was reduced to 1 × 2 mm^2^, and a parallel plate collimator with the angular acceptance of 0.35° was inserted into the diffracted beam in order to decrease the effect of defocusing. A JEOL JEM 1200 EX transmission electron microscope was used for TEM analysis. A plane view specimen was prepared by mechanical grinding and polishing of a sample from its substrate side followed by Ar ion milling on a liquid nitrogen-cooled holder. Optical characterization in the range of 240–900 nm was performed by the USB4000 spectrometer from Ocean Optics.

Thermal stability of Ga_2_O_3_ films was monitored in situ by the XRD while applying the high-temperature annealing cycle using the Anton Paar DHS1100 domed hot stage annealing chamber under vacuum (>1 mBar). Consecutive symmetrical 2θ/ω scans were performed at each annealing step within the temperature range ramping up from ~31 °C to 1100 °C and back down to ~66 °C using 50 °C increments. Time between the measurement was kept close to 1 min to allow temperature stabilization. Surface morphology was investigated by the NT-MDT NTEGRA Prima atomic force microscope (AFM) in tapping mode. Resistivity of the films was examined by room-temperature Van der Pauw method. Despite of the Si doping, the films were found to be highly resistive, similar to previous reports [31].

## 3. Results

### 3.1. Crystal Structure of ε/κ-Ga_2_O_3_ Thin Films

**Figure 1:**
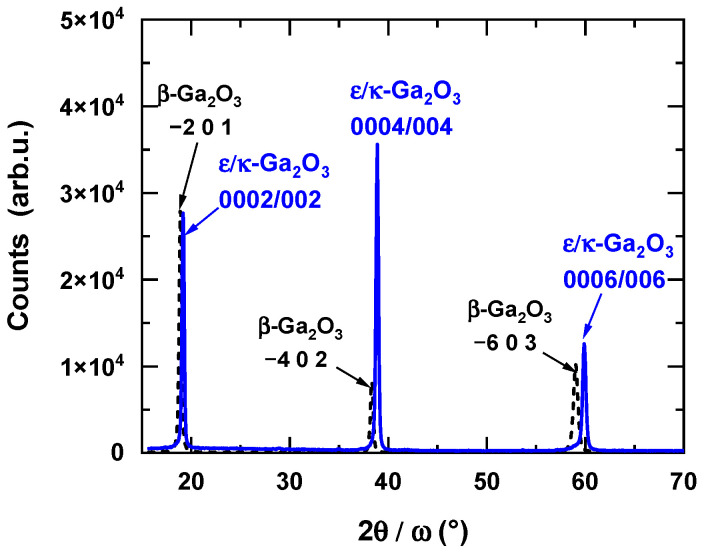
Comparison of symmetric 2θ/ω scans of β-phase and ε/κ-phase Ga_2_O_3_ films grown by LI-MOCVD. Details on the growth of β-Ga_2_O_3_ films can be found in Ref. [27].

For thin films with strong preferred orientation, only diffractions from one family of lattice planes can be observed in symmetrical 2*θ/ω* diffraction patterns. This is the case of the monoclinic *β*-Ga_2_O_3_ layer grown on *c*-sapphire, where the wide angle diffraction pattern exemplified in Figure 1 [27] contains only the diffractions $\bar{2}01$, $\bar{4}02$, and $\bar{6}03$ at the 2*θ* angles 18.907°, 38.388°, and 59.091°, respectively (indexing according to PDF 00-041-1103). Similar diffraction patterns are produced either by *c*-oriented hexagonal *ε*-Ga_2_O_3_ or by *c*-oriented orthorhombic *κ*-Ga_2_O_3_. The 2*θ* angles of the diffractions 0002, 0004, and 0006 are 19.164°, 38.892°, and 59.918°, respectively, for *ε*-Ga_2_O_3_ (ICSD 236278) and 19.105°, 38.769°, and 59.717°, respectively, for κ-Ga_2_O_3_ (ICSD 14747). Identification of the phases can be conducted by careful determination of the position of the diffraction maxima by standard X-ray evaluation software. In this way, the monoclinic *β* phase can be easily distinguished from *ε* and *κ* phases. However, this straightforward method fails for the resolution of *ε* and *κ* phases. As it is seen from the 2*θ* values listed above, the corresponding diffraction maxima occur almost at the same angles 2*θ*. In this case, the measurement of a *φ* scan of an appropriate diffraction *hkl* inclined by a particular angle *χ* from the sample normal can help to resolve the two phases. In general, for unambiguous identification of a particular phase, it is sufficient to find at least one diffraction that does not coincide with the diffractions of other phases, i.e., the diffraction angles 2*θ* and/or the angle of inclination *χ* of the phase to be identified is well separated from the corresponding angles of the other phases. Unfortunately, this is not the case of the *ε* and *κ* phases of Ga_2_O_3_. Both structures are tightly interconnected and their structures are conformable on an atomic level [20]. The hexagonal Ga_2_O_3_ has higher symmetry and the structure can be described as a smaller unit cell with shorter in-plane lattice parameters. On the other hand, the lower symmetry and larger unit cell of the orthorhombic Ga_2_O_3_ results in a larger number of accessible diffractions. As will be shown below, however, for all measurable diffractions of the *ε* phase, at least one diffraction of the *κ* phase with almost identical 2*θ* and *χ* angles can be found.

In the following, the subscripts *h* (hexagonal) and *o* (orthorhombic) will be used to label the lattice parameters and the diffraction indices of *ε*-Ga_2_O_3_ and *κ*-Ga_2_O_3_, respectively. Comparing the in-plane lattice parameters *a_h_* = 0.29036 nm, *a_o_* = 0.50463 nm, and *b_o_* = 0.87020 nm of *ε* and *κ* phases of Ga_2_O_3_, respectively, one can reveal the following relations:

(1) $$a_{o}\approx \sqrt{3}a_{h}\;\;\;b_{o}\approx 3a_{h}$$

**Figure 2:**
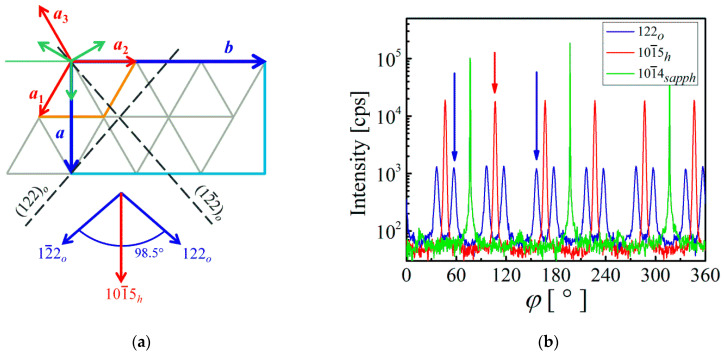
(**a**) Schematic drawing of the coincidence of the *ε*-Ga_2_O_3_, *κ*-Ga_2_O_3,_ and the *c*-sapphire lattices. The crystallographic axes of hexagonal and orthorhombic Ga_2_O_3_ within the plane (0001) of sapphire substrate are shown as blue and red arrows, respectively. The green arrows are the hexagonal axes of sapphire substrate. Black dashed lines represent the intersections of the planes ${\left(122\right)}_{o}$ and ${\left(1\bar{2}2\right)}_{o}$ of the orthorhombic lattice with the plane of interface. The blue and red arrows in the lower part of the figure are the projections of three particular diffraction vectors into the plane of interface; (**b**) *φ* scans of the selected diffractions of orthorhombic Ga_2_O_3_ (blue line), hexagonal Ga_2_O_3_ (red line), and sapphire (green line). Three selected maxima that correspond to diffraction vectors in (**a**) are marked by blue and red arrows, respectively. Note that the hexagonal Ga_2_O_3_ lattice is rotated by 30° with respect to the sapphire lattice.

Both lattices almost perfectly coincide if the orientations of their base vectors are chosen as shown in Figure 2a. Red and blue arrows represent the basic translation vectors of hexagonal and orthorhombic lattices, respectively. The translation vector magnitudes depicted in Figure 2a can be expressed as $a_{h}=\left|a_{1}\right|=\left|a_{2}\right|=\left|a_{3}\right|$, $a_{o}=\left|a\right|,$ and $b_{o}=\left|b\right|$. The projections of the unit cells into the (0001) plane of the sapphire substrate are drawn as an orange rhombus and light blue rectangle. For completeness, the orientations of the hexagonal in-plane axes of sapphire substrate are also shown schematically as green arrows. The relations between the hexagonal and orthorhombic base vectors can be then written in vector form as

$$a\approx 2a_{1}+a_{2}$$

(2) $$b\approx 3a_{2}$$

$$c_{o}\approx c_{h}$$

where a, b and $a_{1}$, $a_{2}$ are the in-plane base vectors of orthorhombic and hexagonal phases, respectively, and $c_{o}$ and $c_{h}$ are the corresponding vectors in the *c* direction. The coefficients at $a_{1}$, $a_{2}$ and $c_{h}$ in Equation (1) can be arranged into the form of a matrix. The diffraction indices $hkl_{o}$ and $hkil_{h}$ of orthorhombic and hexagonal lattices are then related through this transformation matrix according to the relation:

(3) $${\left(\begin{array}{c} h\\ k\\ l \end{array}\right)}_{o}=\left(\begin{array}{ccc} 2 & 1 & 0\\ 0 & 3 & 0\\ 0 & 0 & 1 \end{array}\right){\left(\begin{array}{c} h\\ k\\ l \end{array}\right)}_{h}$$

Note that the third index *i* of the hexagonal notation is omitted in Equation (3). Using this formula, one can easily find the indices of diffractions of the orthorhombic phase that are equivalent to those of the hexagonal phase. Careful and systematic inspection of the calculated diffraction pattern of *ε*-Ga_2_O_3_ (see e.g., ICSD 236278) reveals that all diffractions that are suitable for *φ* scans (diffractions excepting *hki*0 and 000*l*) have their counterparts among the diffractions of *κ*-Ga_2_O_3_.

For illustration, three diffractions of *ε*-Ga_2_O_3_, namely $10\bar{1}1_{h}$, $10\bar{1}5_{h}$, and $11\bar{2}4_{h}$ are listed in Table 1, along with their diffraction angle 2*θ*, inclination angle *χ,* and the calculated modulus squared structure factors ${\left|F\right|}^{2}$. It is interesting to note that each diffraction in the hexagonal phase has two different counterparts in the orthorhombic phase. It can be seen that, e.g., the diffractions $10\bar{1}1_{h}$ and $01\bar{1}1_{h}$ are equivalent in hexagonal phase but the diffractions $201_{o}$ and $131_{o}$ of orthorhombic phase are not. Although their angular parameters 2*θ* and *χ* are almost identical, the intensities differ significantly. This stems from a different symmetry of both structures. From a practical point of view, the most important result is that there are always three diffractions, e.g., $10\bar{1}5_{h}$, $205_{o}$, and $135_{o}$, which can contribute to the same *φ* scan. In addition, in the layers grown on *c*-sapphire substrates, three orientation variants of *κ*-Ga_2_O_3_ lattices rotated by 120° and 240° are usually developed as a consequence of the symmetry of the (0001) surface. Therefore, both diffractions $205_{o}$ and $135_{o}$ of orthorhombic phase contribute equally to all six maxima observed in the *φ* scan. The difference in the intensities of $205_{o}$ and $135_{o}$ diffractions is then cancelled and all observed maxima have approximately the same intensities. This can be seen in Figure 2b, where a typical *φ* scan with six pronounced maxima is shown (red curve). The curve was recorded with the angular parameters of the diffraction $10\bar{1}5_{h}$, i.e., 2*θ* = 62.280° and *χ* = 36.36°, but it is highly probable that the diffractions $205_{o}$ and $135_{o}$ of orthorhombic phase contribute to the observed maxima as well. An important conclusion from the above considerations is that the presence or absence of *ε*-Ga_2_O_3_ cannot be proven by measuring the *φ* scans of diffractions of the hexagonal phase. All *φ* scans can be equally well interpreted within the framework of the orthorhombic *κ*-Ga_2_O_3_ phase.

The identification of the *κ* phase in Ga_2_O_3_ thin films is more useful. One can find several diffractions of the orthorhombic *κ*-Ga_2_O_3_ phase that are suitable for *φ* scans and that do not have a counterpart among the diffractions of the hexagonal phase. It is easy to see that the diffraction $122_{o}$ fulfils these criteria (see Figure 2a). Inverting the transformation Equation (2), one obtains non-integer indices $\frac{1}{6}\frac{2}{3}2_{h}$ (or more precisely $\frac{1}{6}\frac{2}{3}\bar{\frac{5}{6}}2_{h}$) that are forbidden for standard diffractions, i.e., the corresponding diffraction for the hexagonal phase does not exist. The intersections of two lattice planes ${\left(122\right)}_{o}$ and ${\left(1\bar{2}2\right)}_{o}$ of the orthorhombic phase with the (0001) plane of the sapphire substrate are schematically shown in Figure 2a by black dashed lines. The projections of their diffraction vectors $122_{o}$ and $1\bar{2}2_{o}$ are shown in the lower part of the figure by blue arrows along with the projection of the diffraction $10\bar{1}5_{h}$ of the hexagonal phase (red arrow). The angle 98.5° between them can be calculated from the lattice parameters of the *κ* phase. Due to the symmetry of the orthorhombic lattice, one orientation variant contributes with four maxima to the *φ* scan with alternating angular distances 98.5° and 81.5°. Considering three orientation variants, the expected total number of maxima is twelve. This is confirmed in Figure 2b, where the *φ* scan of $122_{o}$ diffraction measured with 2*θ* = 33.345° and *χ* = 54.63° is shown by the blue curve. Three selected maxima of *φ* scans that correspond to diffraction vectors depicted in Figure 2a are marked by corresponding blue and red arrows in the *φ* scan (Figure 2b).

From the above analysis, we can conclude that the *φ* scan of the diffraction $122_{o}$ can serve as an indicator of the presence of orthorhombic *κ*-Ga_2_O_3_ phase in the layer. It is worth noting that appearance of $122_{o}$ maxima in the *φ* scan also implies the existence of maxima in the $10\bar{1}5_{h}$ *φ* scan due to diffractions $205_{o}$ and $135_{o}$, regardless of the ε-phase presence. These diffractions overlap possible contribution of the hexagonal diffractions $10\bar{1}5_{h}$, preventing the unambiguous identification of the hexagonal Ga_2_O_3_ phase. The only possibility to detect the ε-phase is a complete suppression of the κ-phase, i.e., the Ga_2_O_3_ layer has to be grown single-phased. In this case, the maxima are detected only in the $10\bar{1}5_{h}$ *φ* scan, while no maxima could be detected in the $122_{o}$ *φ* scan.

Finally, from the recorded *φ* scans depicted in Figure 2b and from the orientation of the base vectors depicted in Figure 2a, we can establish the orientation relationships between the *ε*-Ga_2_O_3_, *κ*-Ga_2_O_3,_ and the *c*-sapphire substrate as

$$\varepsilon -{\mathrm{Ga}}_{2}O_{3}\left(0001\right)\left[10\bar{1}0\right]║\kappa -{\mathrm{Ga}}_{2}O_{3}\left(001\right)\left[100\right]\;\varepsilon -{\mathrm{Ga}}_{2}O_{3}\left(0001\right)\left[10\bar{1}0\right]║\mathrm{sapphire}\left(0001\right)\left[2\bar{1}\bar{1}0\right]\;\kappa -{\mathrm{Ga}}_{2}O_{3}\left(001\right)\left[100\right]║\mathrm{sapphire}\left(0001\right)\left[2\bar{1}\bar{1}0\right].$$

**Figure 3:**
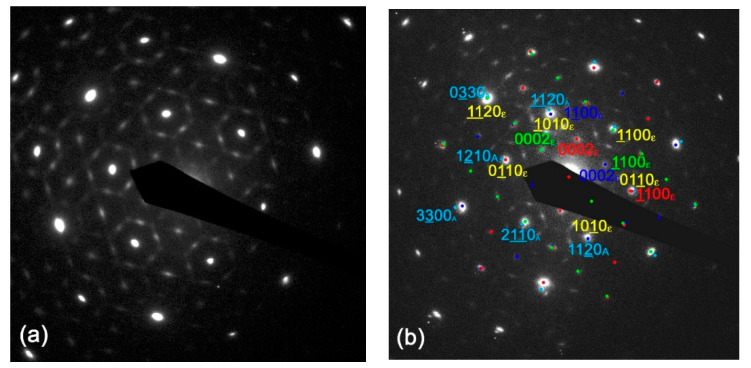

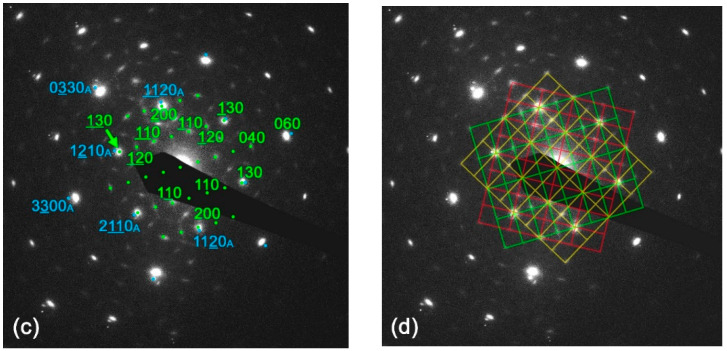
(**a**) Plan-view SAED pattern taken from a thin part of specimen without the substrate contribution. (**b**) SAED pattern from a thicker part of specimen shows possible indexing using pure ε-phase presence. The indices A and ε correspond to Al_2_O_3_ and Ga_2_O_3_ diffractions, respectively. (**c**) The green indices correspond to one possible domain orientation of the κ-phase. (**d**) The schema shows how the presence of the κ- phase domains rotated by 60° and 120° can explain all diffraction spots of the pattern.

In the case of plan-view TEM, electron diffraction showed a rather complex pattern (Figure 3a). The SAED pattern shown in Figure 3a was taken from a thin part of the specimen, thus no contribution from the substrate (including any possible double diffraction substrate–layers spots) were involved here. For analysis of the epitaxial relation, a pattern from the thick part of the specimen was used (Figure 3b–d). To describe the pattern using the pure hexagonal ε- phase, one should suppose the layers were composed of domains with seven different domain orientations. One with the epitaxial relation ε-Ga_2_O_3_ (0001) $10\bar{1}0$ || Al_2_O_3_ (0001) $\left[11\bar{2}0\right]$ and six with the epitaxial orientations ε-Ga_2_O_3_ $\left(11\bar{2}0\right)$ 0002|| Al_2_O_3_ (0001) $\langle 11\bar{2}0\rangle$. In this way, one can describe only principal high intensity diffraction spots of the SAED pattern, while the other could be possibly explained by a presence of special lattice ordering in the domains. However, such explanation is a blind alley, because of the presence of the six orientations of $\left(11\bar{2}0\right)$ ε-Ga_2_O_3_. However, this was not reflected in the layer XRD analysis, where only 0002 diffractions were observed on 2θ-ω scans. Thus, the diffraction pattern cannot be explained by the pure ε-Ga_2_O_3_ phase.

Instead, the experimental SAED pattern can be fully explained by presence of pure orthorhombic κ-phase. Figure 3c shows indexation of one of the possible orientations of (001) oriented κ-Ga_2_O_3_ and its relation to the Al_2_O_3_ substrate (its diffraction spots are indexed by blue colour numbers with index A). A combination of three domain orientations of (001) κ-Ga_2_O_3_ mutually rotated by 60° can explain all observed diffraction spots in the pattern (Figure 3d). Because the κ-phase is not centrosymmetric, one can await six possible domain orientations. Yet, similarly to the XRD analysis, it is not possible to exclude the ε-Ga_2_O_3_ phase presence from the Ga_2_O_3_ layer, because all potentially possible diffraction spots belonging to the (0001) ε-Ga_2_O_3_ phase would be superposed to the intense diffraction spots from the three (001) κ-Ga_2_O_3_ domain orientation. A possible way to improve the crystal quality of our films may be the LI-MOCVD growth on a vicinal sapphire substrate with an intentional miss-cut angle from the (0001) surface. A similar approach was also applied for Ga_2_O_3_ epitaxy using standard MOCVD [32].

### 3.2. Transmittance Spectrum

**Figure 4:**
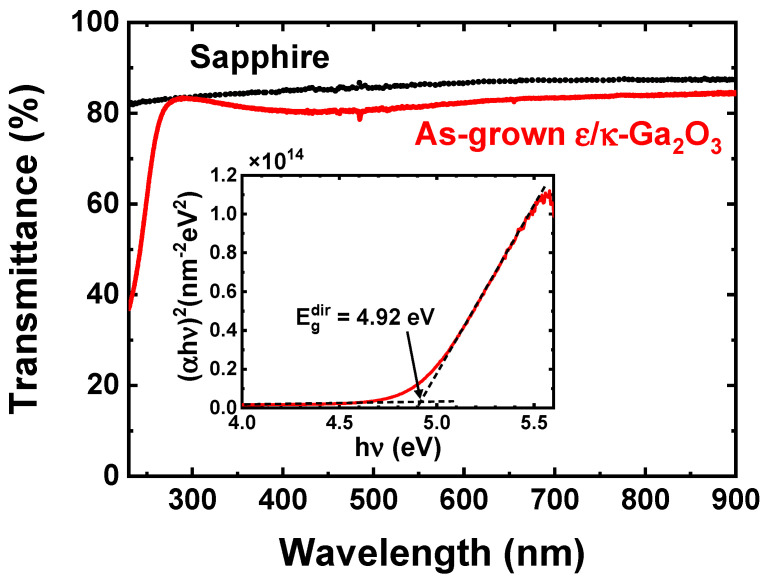
Transmittance spectra of ε/κ-Ga_2_O_3_ thin film on a sapphire substrate and bare sapphire substrate. Inset shows a Tauc plot for the direct optical transition of ε/κ-Ga_2_O_3_ thin film.

Figure 4 shows the UV–VIS transmittance spectra of ε/κ-Ga_2_O_3_ thin film on sapphire substrate and the transmittance spectrum of the bare sapphire substrate as a reference. A high optical transmittance of the ε/κ-Ga_2_O_3_ thin film is observed in the region of 300–900 nm wavelengths. The optical absorption edge of the ε/κ-Ga_2_O_3_ thin film in the UV part of the spectrum indicates a high value of the optical band gap. The Tauc relation between absorption coefficient (α) and photon energy (*hυ*) is defined as [33]

(4) $$\alpha hv=A{\left(hv-E_{g}\right)}^{n}$$

which was further used to construct Tauc plot and to analyze the optical band gap (*E_g_*) of the prepared layer (inset of Figure 4). In Equation (4), *A* is a constant, and *n* is the power factor with values of 0.5 or 2 associated with a direct or indirect optical transition, respectively [33]. Linear behavior of the (*αhυ*)1/*n* vs. *hυ* Tauc curve with *n* equal to 0.5 revealed a direct optical transition in the ε/κ-Ga_2_O_3_ film. The extracted value of the direct optical bandgap is *E_g_* = 4.92 eV, which is in good agreement with published values 4.9–5.02 eV for κ-Ga_2_O_3_ [34,35] and 4.89 –5.0 eV for ε-Ga_2_O_3_ films [36,37].

### 3.3. Thermal Stability of ε/κ-Ga_2_O_3_ Thin Films

**Figure 5:**
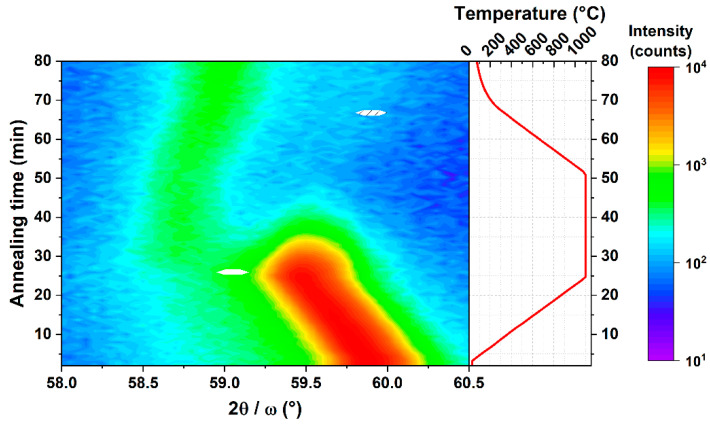
Time evolution of the in situ XRD symmetric 2θ/ω scans monitored during the high-temperature annealing heating/cooling cycle up to 1100 °C. The right panel shows the temperature evolution.

**Figure 6:**
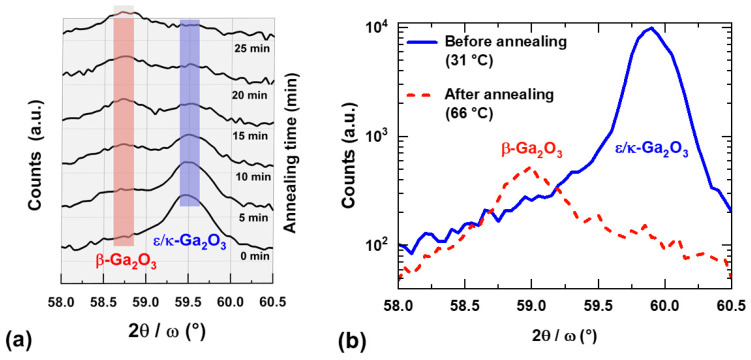
(**a**) Detailed time evolution of the in situ XRD symmetric 2θ/ω scans acquired at the annealing temperature of 1100 °C. (**b**) 2θ/ω scans of the films before and after the high-temperature annealing cycle shown in Figure 4.

Thermal stability of the prepared ε/κ-Ga_2_O_3_ films was first investigated by a high-temperature heating/cooling cycle using in situ XRD 2θ/ω measurements. Figure 5 shows evolution of the ε/κ-Ga_2_O_3_ 0006 diffraction (59.87° at 30 °C) of the film heated from 31 up to 1100 °C followed by a 25-minute long dwelling time and cooling down to 66 °C in vacuum. It can be inferred that ε/κ-phase is stable up to 1100 °C for the given temperature ramp rate, while it starts to degrade after ~5 min upon 1100 °C exposure. Note the shift in the 0006 diffraction toward lower angles during heating corresponds to thermal expansion of the lattice. The onset of the degradation is highlighted in Figure 6a, showing detail time evolution of the XRD pattern at annealing temperature of 1100 °C. For annealing time >5 min, the intensity of ε/κ-Ga_2_O_3_ 0006 diffraction starts to gradually decrease and diminishes after 20 min of annealing. Instead, the $\bar{6}03$ diffraction of β-Ga_2_O_3_ (~59°) with a much weaker intensity compared to the ε/κ-phase evolves after 10 min of the annealing and its intensity remains stable for the rest of the dwelling time as well as the cooling cycle. It is also interesting to compare the XRD patterns measured before and after the heating cycle shown in Figure 6b. These data confirm clear degradation of the ε/κ-phase and its partial recrystallisation to β-Ga_2_O_3_. In addition, a strong decrease in the XRD peak intensity between the as-deposited ε/κ-phase and degraded β-phase (by factor of ~20) suggests a strong deterioration in the crystalline quality, also indicating notable amorphization of the degraded films.

**Figure 7:**
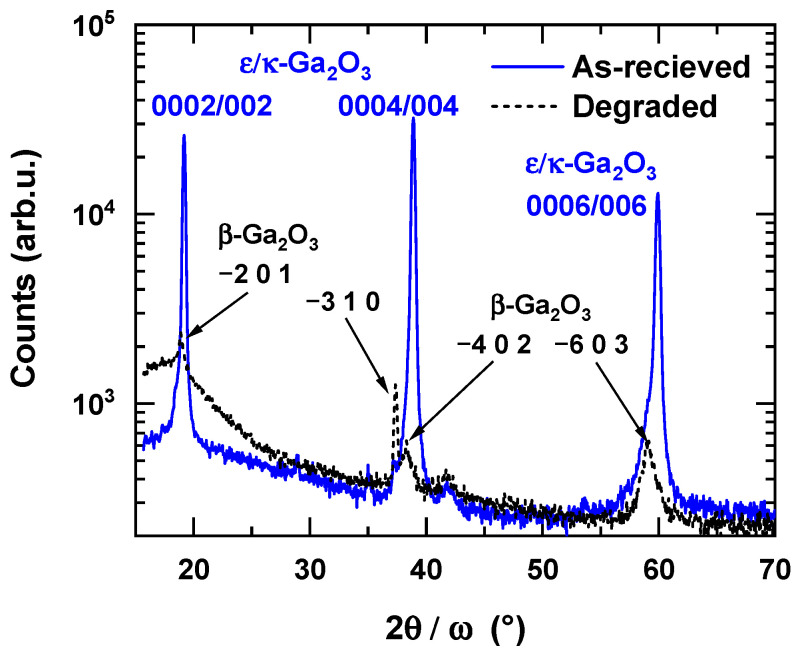
Typical 2θ/ω scans of the as-grown ε/κ-Ga_2_O_3_ films and the degraded films annealed at 900 °C for 30 min.

Based on the relatively fast onset of the film degradation annealed at 1100 °C, we performed prolonged annealing experiments at lower temperatures. As-deposited films were annealed at 700, 800, and 900 °C in a vacuum successively for 10, 20, and 40 min and were analyzed using ex situ XRD and AFM after each annealing step. The XRD results (summarized in Table 2) show that ε/κ-Ga_2_O_3_ films remain stable during annealing at 700 and 800 °C for the entire annealing time examined. Samples annealed at 900 °C remained stable after the first annealing cycle (10 min), but degraded after the second annealing cycle (30 min cumulative time). The 2θ/ω scans measured before and after the second annealing cycle at 900 °C, shown in Figure 7, suggest that the film was transformed to β-Ga_2_O_3_ with two dominant crystal orientations, namely $\bar{2}01$ and $\bar{3}01$. Similar to the high-temperature experiments, a strong drop in the diffraction peak intensity between the as-deposited and degraded films was observed (also by a factor of ~20), indicating possible amorphization of the degraded films.

**Figure 8:**
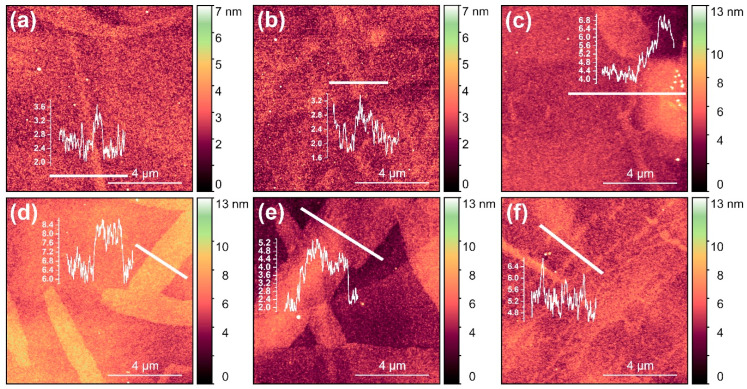
AFM-resolved surface topography of as-grown ε/κ-Ga2O3 films (**a**) and the films subjected to annealing at 800 °C for 10 min (**b**), 30 min (**c**), and 70 min, (**d**) and at 900 °C for 10 min (**e**) and 30 min (**f**) cumulative time. The insets show the AFM scans along the solid white lines.

AFM was used to investigate the surface morphology of the as-grown, annealed, as well as degraded, thin films. Figure 8 shows typical surface morphology of as-grown ε/κ-Ga_2_O_3_ layers (a), films annealed at 800 °C for 10 (b), 30 (c), and 70 min (d), and films annealed at 900 °C for 10 (e) and 30 min (f), i.e., degraded film. Additionally shown are selected AFM line scans performed along the solid white lines to better visualize the surface features formed during the annealing cycle. As-grown samples showed very smooth surface with root mean square (RMS) roughness ~0.8 nm. Small line-shaped features (width of 50–200 nm) with a height of ~1 nm can be observed on the surface. For a sample annealed at 800 °C, negligible changes in surface morphology were observed after 10 min of annealing time (Figure 8b). The prolongation of annealing time resulted in notable increase in the RMS roughness to 1.2–1.3 nm, which can be attributed to emergence of well-recognizable surface features for annealing time >10 min, having the step height of about 2 nm. Similar features were also formed for films annealed at 900 °C for 10 min, i.e., the non-degraded sample with ε/κ-phase. The height increased to about 3 nm. Increasing in the surface roughness with annealing time and temperature before film degradation can result from localized loss of oxygen, e.g., from interstitial sites or surface contaminants degasification.

Interestingly, after degradation of the ε/κ-phase during annealing at 900 °C for 30 min (Figure 8f), the film became smoother (RMS roughness of ~1 nm) as compared to previous annealing. On the other hand, clear change in surface morphology was observed, where the surface striation can be inferred from the AFM image (Figure 8f). Based on the XRD results (Figure 7), the possible explanation of this effect can be predominant amorphization of the film and the partial recrystallization of epitaxial ε/κ into the polycrystalline β-Ga_2_O_3_ phase.

## 4. Discussion

The thermal stability of ε/κ-Ga_2_O_3_ films grown by MOCVD were studied by Xia et al. [14] and Fornari et al. [28] in N_2_ and N_2_ or O_2_ atmosphere, respectively. Xia et al. [14] reported ε/κ-phase stability up to 800 °C for furnace annealing in N_2_ for 30 min. A mixture of ε/κ-phase and β-phase was observed after annealing at 850 °C for 30 min and the films eventually transformed completely to pure β-phase when subjected to annealing at 900 °C. Fornari et al. [28] found similar behavior, where the XRD results suggest thermally stable films after 3-hour long annealing at 700 °C in N_2_ as well as O_2_ atmosphere, while complete conversion to pure β-phase with deteriorated crystal quality took place after annealing at 900 °C. Interestingly, 3-hour long annealing at 800 °C led to amorphization of the film, which was attributed to an intermediate disordered step of the crystal structure conversion. Finally, a detailed study using in situ differential scanning calorimetry revealed that the initial phase transformation took place already at 650 °C.

Our results are fully in line with the previous studies, extending the thermal stability of ε/κ-Ga_2_O_3_ studies also in vacuum ambient. In particular, it clearly demonstrates that the thermal budget (i.e. high temperature applied during certain time) rather than temperature itself is important in assessing the thermal stability of an epitaxial film with metastable crystal structure. While our film retained its structure only for 5 min at 1100 °C and 10 min at 900 °C, it can be expected that prolonged annealing at 800 °C would also lead to phase conversion. Further, vacuum annealing represents somewhat of a specific condition for thermal stability of metal-oxide films, as degasification of the film can occur. This can explain much lower crystal quality of the β-phase Ga_2_O_3_ after phase transformation and strong amorphization of the film as compared to those reported previously [14,28]. It is also worth mentioning that thickness of the epitaxial film may affect its thermal stability. This was observed for α-Ga_2_O_3_ MOCVD films, where thinner films show the onset of phase transformation/thermal degradation at higher temperatures compared to thicker films [38]. This was attributed to the thermal stress caused by the difference in the lattice thermal expansion coefficient of α-Ga_2_O_3_ and sapphire substrate, where the thicker films reach the critical stress level at lower annealing temperatures than the thinner films. A similar effect can be expected to take place also for ε/κ-Ga_2_O_3_.

## 5. Conclusions

In summary, Si-doped ε/κ-Ga_2_O_3_ layers were grown on c-plane sapphire using LI-MOCVD. As deduced from XRD and TEM, highly ordered films with several orientation variants of ε/κ-Ga_2_O_3_ lattices were grown and the orientation relationships between the two phases and the c-sapphire substrate was established. It was demonstrated that the presence or absence of ε-Ga_2_O_3_ cannot be proved by measuring the φ scans of diffractions of the hexagonal phase. All φ scans can be equally well interpreted within the framework of the orthorhombic κ-Ga_2_O_3_ phase. On the other hand, a single-phased hexagonal phase can be identified by XRD, if the hexagonal maxima are detected only in a $10\bar{1}5_{h}$ φ scan, while no maxima are detected in the orthorhombic $122_{o}$ φ scan. Similarly, electron diffraction in conventional plan-view TEM can clearly identify the presence of a κ-Ga_2_O_3_ phase by a more complex SAED pattern in comparison to one from the ε-Ga_2_O_3_ phase. However, this method cannot exclude the ε-Ga_2_O_3_ presence if six possible domain orientations (or minimally three of them) of the κ-Ga_2_O_3_ phase are present in the sample. The prepared films show enhanced thermal stability; layer degradation via partial phase conversion to β-Ga_2_O_3_ and possible amorphization of the film was observed after ~5-min long annealing at 1100 °C, or 10-min annealing at 900 °C. These results are very promising and open new possibilities, e.g., towards growth of various III-N barrier layers on the Ga_2_O_3_ channel layer for processing of heterostructure FETs.

## Figures and Tables

**Table 1:** Three selected diffractions of hexagonal *ε*-Ga_2_O_3_ (left panel) and their counterparts in orthorhombic *κ*-Ga_2_O_3_ (right panel). 2*θ* is the diffraction angle, *χ* is the inclination angle with respect to the sample normal, and ${\left|F\right|}^{2}$ is the calculated modulus squared structure factor.

Hexagonal *ε*-Ga_2_O_3_				Orthorhombic *κ*-Ga_2_O_3_			
$hkil_{h}$	2*θ* [°]	*χ* [°]	${\left|F\right|}^{2}$[--]	$hkl_{o}$	2*θ* [°]	*χ* [°]	${\left|F\right|}^{2}$ [--]
$10\bar{1}1_{h}$	37.046	74.80	650	$201_{o}$	36.917	74.79	23,570
$01\bar{1}1_{h}$	37.046	74.80	650	$131_{o}$	37.035	74.84	29,758
$10\bar{1}5_{h}$	62.280	36.36	2478	$205_{o}$	62.063	36.35	68,954
$01\bar{1}5_{h}$	62.280	36.36	2478	$135_{o}$	62.143	36.44	80,454
$11\bar{2}4_{h}$	77.638	57.89	323	$334_{o}$	77.409	57.91	12,260
$\;\;\;\bar{1}2\bar{1}4_{h}$	77.638	57.89	323	$064_{o}$	77.627	58.00	6751

**Table 2:** Summary of annealing conditions and their influence on the degradation of ε/κ-Ga_2_O_3_ grown on sapphire using LI-MOCVD.

Annealing Ambient	Annealing Temperature (°C)	Cumulative Annealing Time (min)		
		10	30	70
Vacuum	700	ε/κ	ε/κ	ε/κ
	800	ε/κ	ε/κ	ε/κ
	900	ε/κ	deg.	deg.
